# An exploratory cluster randomised trial of a university halls of residence based social norms intervention in Wales, UK

**DOI:** 10.1186/1471-2458-12-186

**Published:** 2012-03-13

**Authors:** Simon Murphy, Graham Moore, Annie Williams, Laurence Moore

**Affiliations:** 1 DECIPHer, Cardiff School of Social Sciences, Cardiff University, 1-3 Museum Place, Cardiff CF10 3BD, UK

## Abstract

**Background:**

Excessive alcohol consumption amongst university students has received increasing attention. A social norms approach to reducing drinking behaviours has met with some success in the USA. Such an approach is based on the assumption that student's perceptions of the norms of their peers are highly influential, but that these perceptions are often incorrect. Social norms interventions therefore aim to correct these inaccurate perceptions, and in turn, to change behaviours. However, UK studies are scarce and it is increasingly recognised that social norm interventions need to be supported by socio ecological approaches that address the wider determinants of behaviour.

**Objectives:**

To describe the research design for an exploratory trial examining the acceptability, hypothesised process of change and implementation of a social norm marketing campaign designed to correct misperceptions of normative alcohol use and reduce levels of misuse, implemented alongside a university wide alcohol harm reduction toolkit. It also assesses the feasibility of a potential large scale effectiveness trial by providing key trial design parameters including randomisation, recruitment and retention, contamination, data collection methods, outcome measures and intracluster correlations.

**Methods/design:**

The study adopts an exploratory cluster randomised controlled trial design with halls of residence as the unit of allocation, and a nested mixed methods process evaluation. Four Welsh (UK) universities participated in the study, with residence hall managers consenting to implementation of the trial in 50 university owned campus based halls of residence. Consenting halls were randomised to either a phased multi channel social norm marketing campaign addressing normative discrepancies (n = 25 intervention) or normal practice (n = 25 control). The primary outcome is alcohol consumption (units per week) measured using the Daily Drinking Questionnaire. Secondary outcomes assess frequency of alcohol consumption, higher risk drinking, alcohol related problems and change in perceptions of alcohol-related descriptive and injunctive norms. Data will be collected for all 50 halls at 4 months follow up through a cross-sectional on line and postal survey of approximately 4000 first year students. The process evaluation will explore the acceptability and implementation of the social norms intervention and toolkit and hypothesised process of change including awareness, receptivity and normative changes.

**Discussion:**

Exploratory trials such as this are essential to inform future definitive trials by providing crucial methodological parameters and guidance on designing and implementing optimum interventions.

**Trial registration number:**

ISRCTN: ISRCTN48556384

## Background

Excessive alcohol consumption among university students has been linked to a range of adverse outcomes, including educational difficulties, psychosocial problems, antisocial behaviours, injuries, risky sexual behaviours and drink driving [1]. In the United Kingdom, alcohol consumption levels amongst university aged adults increased rapidly during the 1990s [2]. Recent studies suggest that just over half of UK university students 'binge drink' (i.e. consume 5 or more drinks in one sitting) at least once per week [3,4], whilst as many as 80% binge drink at least once a month [4]. One recent study estimated average alcohol consumption at 25 units per week for 1^st ^year male UK undergraduates and 16 for 1st year women [5], significantly above current public health recommendations. Recent UK government policy of increasing the percentage of young people going to university has perhaps had the effect of exposing a larger proportion of the population to this high-risk drinking environment.

Alcohol consumption amongst university students has to date proved highly resistant to intervention efforts [6]. One approach, which has shown some promise in experimental studies, is addressing the perceived social norms that are posited to influence alcohol consumption [7]. Perceived norms take the form of descriptive norms, with behaviour modelled through observation of the behaviour of significant others; or injunctive norms, where the individual perceives that their peers expect them to behave a certain way. Interventions underpinned by the social norms approach argue that normative perceptions are highly fallible, with students often overestimating real alcohol consumption patterns among peers [8]. Hence, through providing feedback and correcting misperceptions regarding the behaviours and social expectations of peers, alcohol drinking behaviours may be reduced. Social norms interventions have typically involved provision of mailed, web-based or face-to-face feedback on individual's drinking behaviour and how this compares to norms for their peer group, or social marketing campaigns to promote awareness of actual norms. A recent Cochrane review concluded that feedback-based interventions delivered via the internet or face-to-face on a one-to-one basis appeared to reduce student drinking behaviours, though mailed or group feedback were less effective, and findings for social marketing campaigns were equivocal [7].

Whilst demonstrating promise, such interventions have typically been examined in isolation from the contexts in which they operate and significant questions remain to be addressed regarding how they might be applied in practice. No such studies have taken place in Wales, with the limited number of UK based studies suffering substantial weaknesses such as high levels of attrition [9]. Furthermore, universities are complex systems, whose overall ethos, policies and practices may provide a context supportive of change, or of maintaining the status quo [10]. Interventions which aim to achieve long-term change through simply targeting cognitive factors such as normative perceptions, without addressing the characteristics of the setting which support the status quo are likely to fail in the longer term [11]. Some community-based interventions to reduce alcohol consumption in adolescents have for example been shown to be more effective in rural settings than urban settings, where impacts of the intervention are perhaps drowned out by the multitude of pro-alcohol stimuli in the urban environment [12]. In Welsh universities, university managed accommodation blocks (halls of residence) primarily house students in their first year of attendance, with approximately half of students living in halls during their first year. Given that first year students are at greatest risk of excessive alcohol consumption [5], halls of residence offer potential as a means of reaching those students most at risk for alcohol related intervention.

The proposed research therefore aims to assess the value of a social marketing-led social norms-based intervention implemented in University halls of residence across four Universities in Wales. A survey of first year students was conducted in participating universities in May 2011, in order to establish levels of drinking and the prevalence of alcohol related consequences, as well as normative perceptions. Findings from the survey fed into the development of materials by an Intervention Steering Group to communicate areas of normative misperception (e.g. the extent to which students overestimated peer drinking volume), to be distributed within halls of residence. All halls of residence participating in the study will experience a university-wide alcohol harm reduction toolkit, with half randomised to additionally receive the social norms intervention.

An exploratory cluster randomised design with nested process evaluation will be used to identify appropriate outcome measures and data collection methods, test randomisation processes, assess the extent of contamination across trial arms and establish recruitment and retention rates and intra-cluster correlations to help inform sample size for any future definitive trial. It will also identify whether the intervention effectively mobilises the underpinning theory and that this is sufficient to bring about hypothesised responses in terms of awareness, engagement and changed perception of norms. Whilst intervention acceptability and implementation processes will be assessed within the process evaluation.

## Methods/design

### Research design

**Figure 1:**
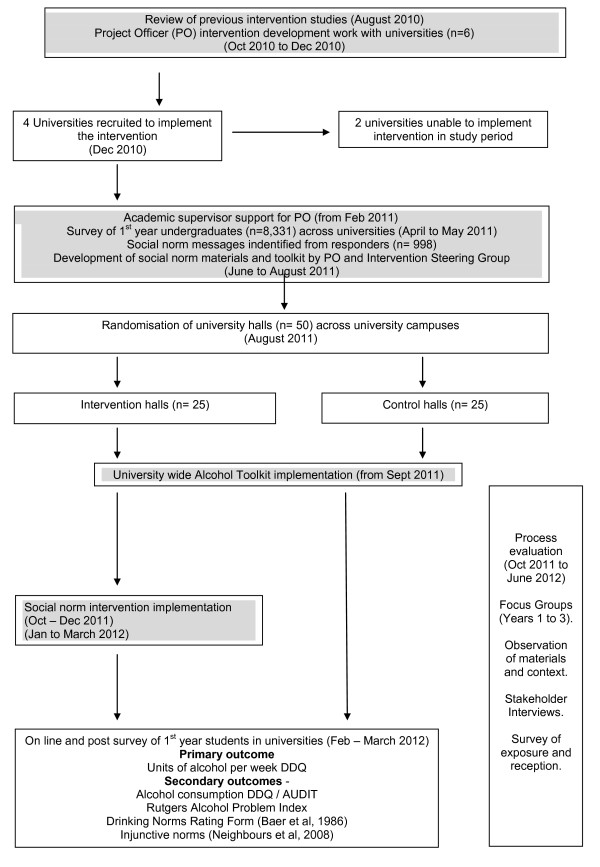
**Research design**.

Figure 1 provides a summary of the study; an exploratory cluster randomised controlled trial design with nested mixed methods process evaluation. Ethical approval was provided by Cardiff University, School of Social Sciences Research Ethics Committee, with separate applications approved for the process evaluation (SREC/752) and the cluster randomised trial (SREC/857)

### The intervention

The intervention is a social norm marketing campaign, which aims to correct misperceptions regarding the behaviours and social expectations of peers and in so doing influence alcohol consumption. The campaign will be delivered in two phases between October 2011 and May 2012 in intervention halls of residence in four universities and will use a variety of materials encompassing posters, beer mats/coasters, leaflets, meal planners and drinking glasses. The campaign will be implemented by university accommodation staff. Social norm messages were based on the results of a survey of first year university students conducted in study universities in late April/May 2011 which identified discrepancies between norms and behaviours. Table 1 highlights the intervention materials and main social norm messages within them.

**Table 1:** Social norms intervention components and examples of core messages communicated within them

Timing	Material	Core message
October 2011	Posters	'Those around you are drinking less than you think: students overestimate what others drink by 44%' 'Most of us significantly overestimate the amount that others drink'
	
	Beer mats/coasters	'Those around you are drinking less than you think: students overestimate what others drink by 44%' 'Most of us significantly overestimate the amount that others drink'
	
	Window stickers	'Few of us approve of people who drink to the point of losing it'

January 2012	Posters	'Most students drink to feel confident, but 70% have embarrassed themselves when drunk'
	
	Drinking glasses	'Time for a break? Many students limit their drinking by including soft drinks in the night'
	
	Gender specific leaflets	Males: '86% of Males have never damaged their halls of residence when drunk' Females: 'How much do you think the average female first year student drinks? Halve it. It really is less than you think.'

Universities in the study also receive a toolkit to promote institutional responsibility for prevention, audit current alcohol misuse policies and practices and which provided advice and guidance on prevention. The toolkit was developed by a National Union of Students (NUS) intervention project officer in consultation with the universities in the study. It was distributed to key university stakeholders in October 2011 with the intention of developing a supportive environment for the intervention. Control halls will be exposed to the toolkit only. The toolkit and social norms intervention were developed collaboratively by Drinkaware, NUS Wales and the Welsh Government following a review of previous interventions and support from an academic supervisor. Their implementation was facilitated by a dedicated NUS project officer. Given the nature of the intervention, it was not possible to blind participants to condition.

### Recruitment

Six universities who had collaborated on the development of the intervention were approached by the evaluation team to participate in the study, with four agreeing to implement the intervention in the study period. Reasons for non-participation were related to difficulties in implementing the intervention and obtaining university consent within the evaluation timeframe. Informed consent for the study was obtained from directors of student services and halls of residence managers. The universities varied in terms of the number of full-time first year students (from 1100 to 3327), and location, with a mixture of urban and rural locations.

### Inclusion/exclusion criteria

The four universities had a total of 51 on campus university owned halls of residence. One female only hall was excluded due to lack of trial arm balance. All remaining halls (n = 50) were eligible for inclusion and consented to randomisation, although 5 halls in one site were empty during the first phase of the campaign due to renovation.

### Randomisation

Blind remote randomisation was used to allocate halls of residence to receive the social norms plus toolkit intervention or toolkit only. Halls were stratified by institution and halls allocated alternately in a list ordered by size, with the group allocation determined by one random number within each stratum.

### Measures

#### Primary outcome

#### Units consumed per week - daily drinking questionnaire

The primary outcome is alcohol consumption in units per week assessed via the Daily Drinking Questionnaire [13]. The measure asks students for details of a typical week rather than exact quantities for the last 7 days, in order to ensure that it reflects habitual drinking. The DDQ has emerged as a favoured measure within RCTs with students due to its brevity, its convergent validity with more laborious drinking measures [13], acceptable internal consistency (Neighbors et al. 2002), good 2-month test-retest reliability for volume and adequate test-retest reliability for frequency [14]) and established ability to detect post-intervention changes. Importantly, the measure also provides comparable estimates regardless of whether administered via the internet or as a pen and paper exercise [15]

### Secondary outcomes

#### Weekly alcohol consumption behaviours - daily drinking questionnaire

Responses can also provide a measure of i) number of days per week drinking in a typical week, ii) number of units per sitting and iv) number of heavy drinking episodes per week.

#### [***Prevalence of higher risk drinking - AUDIT***

The consumption subscale of the Alcohol Use Disorders Identification Tool (AUDIT p15;[16] provides an additional measure of alcohol consumption, allowing estimation of the prevalence of potentially hazardous drinking in control/intervention halls. The scale includes items on frequency of drinking, volume per drinking occasion and frequency of 'binge drinking' (e.g. 8+/6+ units on one occasion for men/women), each scored on a scale of 0-4. In primary care studies, a total summed score of 4 or above for men, or 3 or above for women, has been shown to optimally identify potentially hazardous drinkers [17]

#### Alcohol related consequences - Rutgers alcohol problem index

Secondary outcomes include the 18-item version of the Rutgers Alcohol Problem Index (RAPI) [18]. The index is a well validated measure of alcohol problems with well established psychometric properties among clinical and general population samples ranging from 12 to 21 years. It is commonly used among general university populations in evaluations of alcohol based interventions. All items are typically summed to provide a single continuous variable for alcohol problems, although the factor structure in the current population will be carefully checked.

#### Descriptive norms

In order to assess whether the campaign achieves the hypothesised mechanism of changing perceived descriptive norms for drinking, the evaluation requires a measure of perceived descriptive norms. The drinking norms rating form has been widely used in RCTs and cross sectional studies (Baer et al. 1991) and involves rewording the DDQ to reference others rather than self, therefore having the advantage that perceived normative behaviour is measured in exactly the same way as own behaviour.

#### Injunctive norms

Whilst most previous studies have focused on descriptive norms, many psychological models argue that injunctive norms (i.e. perceived social pressure or social approval) are equally important in shaping behaviour. A scale previously used by Neighbors et al. (2008) was therefore included.

#### Demographics

Measures of gender, age, ethnicity, international/home student status, course studied and place of residence will facilitate an examination of the representativeness of the sample, to assess comparability between groups of students assigned to receive/not receive the social norms intervention and assess potential contamination between trial arms.

#### Acceptability of objective measures

Students will also be asked to indicate whether they would be willing to provide hair samples as an objective measure of alcohol consumption, although it will be made clear that this is a hypothetical question, and that we will not be attempting this at any point in the present study. The question is simply included to evaluate the acceptability of this method among university students if we were to seek funding for a larger definitive trial using more objective measurement approaches in the future.

### Data collection

At four months after initial implementation of the intervention, measures will be collected via a survey to all 1^st ^year university students, offered in web and paper format. They will be recruited via nominated university distribution contacts, who will circulate the link to first year students via email and electronic notice boards between mid-February and the end of March 2012. At least one reminder will be emailed to students during the data collection period. On completion of the questionnaire, data will be captured and processed by a market research company, who will prepare a complete anonymised dataset for analysis. Heads of student services provided consent for the conduct of the survey and students will not be able to complete the survey without completing informed consent tick-boxes. Students will not be asked to provide any identifiable information, other than email addresses, which will be used purely for the purpose of selecting a winner for the £100 prize draw in each university, offered as an incentive for participation. Email addresses will be separated from responses to the web survey and destroyed after the prize draw.

In an attempt to boost student responses, residence hall managers will be asked to promote the survey and the prize draw to residents. To compare the efficacy of two data collection approaches, a paper copy of the questionnaire will be distributed to student halls of residence via accommodation managers, inviting students either to complete the paper copy and return it to the research team in a freepost envelope, or to go to the web-page to complete the survey online. Questionnaires completed in paper form will be returned to the research team in freepost envelopes. These will be stored in a locked cabinet until the web survey data-file is received from the survey company, at which point, questionnaires will be retrieved, data entered into the data-file, and questionnaires returned to the cabinet. Participants will be offered the opportunity to enter a prize draw, with £100 offered to one winner in each participating university by supplying a university email to be kept separately to questionnaire responses. Email addresses will be recorded on a detachable sheet at the start of the questionnaire, which will be separated from the questionnaire once received, with the email address entered into a separate spread sheet and the paper copy destroyed.

### Sample size

Assuming a student response rate of 40%, 1600 completed questionnaires will be available for analysis, an average of 32 students per hall. Assuming an intra-cluster correlation of 0.03, fifty halls of residence will therefore provide 80% power to detect a 0.2 standard deviation difference in units of alcohol consumed using a two-tailed alpha of 0.05.

Assuming a student response rate of 25%, 1000 completed questionnaires will be available for analysis, an average of 20 students per hall. Assuming an intra-cluster correlation of 0.03, fifty halls of residence will therefore provide 80% power to detect a 0.23 standard deviation difference in units of alcohol consumed using a two-tailed alpha of 0.05.

It is not anticipated that the effect size will be of this magnitude, with a much larger trial likely to be necessary to detect realistic effect sizes below 0.1 standard deviation. This study is therefore designed as an exploratory trial to assess the value of the intervention and plan a larger scale study if warranted.

### Process evaluation

Universities are complex systems, whose ethos, policies and practices may provide a context supportive of change, or of maintaining the status quo [10]. Within evaluations of complex interventions, process evaluation is crucial in order to understand what was implemented, how it was received and ultimately, how outcomes were produced. A process evaluation will run alongside the implementation of the programme, throughout the 2011/12 academic year. The process evaluation is concerned with 5 core research questions:

i. What role does alcohol play in students' social life during the transition to university and throughout university life?

ii. How are the toolkit and social norms activities developed and what are their underlying logic models?

iii. How are the toolkit and social norms activities implemented?

iv. How, for whom and in what circumstances, does the toolkit brings about change in university practices

v. How, for whom and in what circumstances does the social norms intervention influence alcohol related beliefs and behaviour?

The process evaluation will encompass 1) group interviews with up to twenty 2^nd ^and 3^rd ^year students in each university focusing upon experiences of alcohol throughout student life, 2) visits by a researcher to each intervention residence hall in order to monitor the distribution and placement of materials, 3) group interviews with up to 6 students in 2 case study halls in each university (one receiving and one not receiving the social norms intervention) exploring awareness and responses to the intervention 4) interviews with stakeholders in each university involved in delivering the intervention. In addition, all residence hall wardens will be asked to complete a brief questionnaire to assess changes in practice over time. Permission will also be requested from university representatives to use routine public data gathered during audits forming part of the toolkit.

Finally, within the survey described above, to assess intervention reach, students will be asked to indicate whether they had seen the intervention materials in their own hall of residence, or in another students' hall of residence. To assess recall, students who recalled seeing any of the norms materials will be asked to identify core messages from a list. Students will also be asked whether messages within the materials were credible and relevant, and whether they felt that exposure to the materials had influenced their normative perceptions or behaviour. These questions will be identical for students in control and intervention halls, allowing assessments of contamination between trial arms. The survey also includes a number of bespoke items from the intervention survey, which informed the social norm intervention, but only where these are linked to specific intervention communications (e.g. some materials focused on round buying behaviour and alternating alcoholic and soft drinks, hence items assessing the prevalence of these behaviours are retained).

Hall of residence managers will be asked for their consent for researchers to visit halls to monitor the placement of campaign materials. Prior to group interviews, an information sheet would be provided, with participants offered the opportunity to ask questions prior to obtaining informed consent. Since part of the process evaluation requires asking different questions of intervention and control premises representatives, the research team members who conduct the process evaluation will be unblinded.

### Analysis

In order to assess exposure to intervention materials and contamination between trial arms, percentages of students within the intervention and control groups reporting having seen each of the intervention materials i) in their own hall of residence and ii) in another students' hall of residence will be examined. Among those students reporting exposure to intervention materials, percentages correctly identifying the messages within them will be calculated for each trial arm. Percentages of students reporting each level of agreement with statements regarding the credibility, relevance and perceived impacts of intervention materials will also be examined for each trial arm.

Whilst the study is likely not sufficiently powered to detect impacts on behaviour, it is likely that relatively large changes in perceived norms will be necessary to produce small changes in behaviour. Hence, regression analyses, with random terms to adjust for clustering at the hall level, and fixed terms to adjust for stratification variables, will examine differences between intervention and control participants in terms of normative perceptions for alcohol consumption and alcohol related consequences. Comparisons between trial arms will be conducted on an intention-to-treat basis. Secondary analyses would compare halls on the basis of researcher observations of whether or not materials were placed.

To inform the design of a potential large scale definitive trial with sufficient power to detect changes in behaviour, intra-cluster correlations and standard deviations will be calculated for total number of units per week. Response rates will be calculated in each trial arm. The percentage of students reporting willingness to provide hair samples will also be presented, whilst among those students reporting that they would only do so if paid, percentages reporting that each level of payment would be required would be presented.

## Discussion

The need to address high levels of alcohol misuse amongst UK student populations has led to a range of possible preventive approaches, including social marketing campaigns that address misperceptions of social norms. However, the lack of a strong evidence base for UK interventions highlights the need for an exploratory trial phase before large scale intervention implementation and the conduct of any definitive trial. Definitive trials require appropriate outcome measures, cost effective data collection, reliable randomisation processes, an understanding of potential contamination across trial arms and a measure of recruitment and retention rates and intra-cluster correlations to help inform sample size calculations. The current study provides the opportunity to generate such information within the context of an exploratory trial of a university halls based social norm marketing intervention. It also provides the opportunity to test the application of the theoretical assumptions underlying the social norm approach by measuring the hypothesised pathways that are posited as leading to behaviour change. These are an assessment of campaign awareness, reception and changes in normative perceptions. The challenges in facilitating such processes with a relatively low intensity interventions informed intervention development and the provision of the supportive environment toolkit and also led to a relatively large sample size for an exploratory trial, to asses such changes in intrapersonal processes. Finally the study provides an important opportunity to assess intervention acceptability and implementation processes to inform optimum intervention content and delivery in any future trial.

## Competing interests

The authors declare that they have no competing interests.

## Authors' contributions

SM, GM and LM were actively involved in the development and design of the study and all authors in the drafting of the manuscript. SM is the principal investigator. GM is co applicant and responsible for the day to day management of the study. LM is co applicant and responsible for statistical oversight of the project. AW is responsible for the conduct of the process evaluation. All authors read and approved the final manuscript.

## Pre-publication history

The pre-publication history for this paper can be accessed here:

http://www.biomedcentral.com/1471-2458/12/186/prepub
